# AI-Enabled Digitalization and Environmental Practices in Municipal Waste Management: Pathways to Climate Sustainability in Pakistan

**DOI:** 10.1007/s00267-026-02573-3

**Published:** 2026-08-04

**Authors:** Muhammad Ahsan Iqbal, Asifa Muhammad Sabir, Kundan Kumar, Md Mahfujul Islam

**Affiliations:** 1 Rawalpindi Women University, Rawalpindi, Pakistan; 2 Sir Syed CASE Institute of Technology, Islamabad, Pakistan; 3 Department of Natural Resource and Society, College of Natural Resources, University of Idaho, Moscow, ID USA

**Keywords:** Municipal waste management, AI-enabled digitalization, Climate sustainability, Environmental practices, Smart public services, Circular economy

## Abstract

Rapid urbanization and the intensifying pressures of climate change have elevated solid waste management (SWM) as a critical public service challenge. Evidence on the joint mediating effects of digital transformation and environmental governance between municipal SWM and climate sustainability remains questionable. This study examines the role of SWM in promoting climate sustainability by evaluating environmental management practices and AI-enabled digitalization. Data were collected from 406 employees across seven Water and Sanitation Service Companies (WSSCs) in Khyber Pakhtunkhwa, Pakistan, using a cross-sectional survey with a structured and validated instrument. The analytical approach involved Confirmatory Factor Analysis (CFA) and Structural Equation Modeling (SEM) with bootstrapped mediation analysis (5000 resamples), executed in SPSS and AMOS software. The study measured waste management effectiveness, environmental practices, AI-enabled digital systems, and sustainability outcomes across economic–environmental–social nexus. Findings reveal a significant positive relationship between effective SWM and climate sustainability (β = 0.41, *p* < 0.001). Environmental practices including regulatory compliance, pollution control, and workforce training along with AI-driven digital capabilities including real-time tracking, route optimization, electronic service reporting, and data dashboards play a strong mediating role (indirect effects: ENV = 0.13; DIG = 0.12; joint = 0.21). The results suggest that municipal waste systems achieve greater climate benefits when routine operations are supported by integrated environmental management and smart digital technologies. The study contributes to the research on sustainable models, circular economy, and smart urban governance, offering policy guidance for municipal authorities in Pakistan, through AI-enabled tools and solutions, environmental governance practices, and SDG-aligned incentives.

## Introduction

The concept of sustainable development has become a major concern of the twenty-first century as governments, organizations, and societies react to the environmental, social, and economic impacts of rapid urbanization and industrial growth (Sachs 2015; IPCC 2022). Solid waste management holds a critical place among the numerous sustainability challenges in developing economies. Poorly managed waste systems lead to environmental pollution, health hazards, and resource loss, thereby restricting progress toward long-term sustainability and climate resilience (Hoornweg and Bhada-Tata 2012; Ferronato and Torretta 2019). Available global evidence shows that waste generation rates are growing at an alarming pace, with developing regions projected to contribute the largest share of future municipal solid waste, placing significant strain on already limited municipal structures (Kaza et al. 2018).

Urbanization and changing consumption patterns have led to a tremendous growth in the quantity and composition of municipal solid waste (MSW) in Pakistan (Guerrero et al. 2013). Inadequate collection coverage, insufficient treatment infrastructure, and weak governance systems have compounded the associated environmental and climate risks. Consequently, national commitments to sustainable cities, responsible consumption, and climate action as defined under the United Nations Sustainable Development Goals (SDGs 11, 12, and 13) have placed solid waste management at their core (United Nations 2022). Solutions to the waste crisis must therefore extend beyond traditional operational approaches toward integrated systems that advance sustainability across economic, environmental, and social dimensions (Schroeder et al. 2019).

The prevailing literature increasingly recognizes that waste management cannot be treated as an isolated municipal activity (Ferronato and Torretta 2019; Zhu et al. 2008). Rather, it must be embedded within broader sustainability frameworks that encompass both environmental stewardship and digital transformation. Environmental practices including regulatory compliance, pollution prevention, and employee training are essential to translating operational waste management improvements into tangible sustainability outcomes (Molina-Azorín et al. 2009; Sarkis et al. 2010). Simultaneously, the emergence of AI-enabled digitalization has opened new possibilities for improving waste management performance through data-driven decision-making, enhanced monitoring, and service optimization (Abdallah et al. 2020; Kumar et al. 2023). Digital tools such as real-time tracking systems, route optimization algorithms, electronic service reporting, and performance dashboards are increasingly deployed to enhance efficiency, transparency, and accountability in municipal services (Al-Thani et al. 2022).

Prior studies have examined environmental management practices and digital transformation as separate influences on sustainability performance in municipal services (Rosa et al. 2020; Zhang et al. 2020). However, these two pathways have not been simultaneously modeled as mediating mechanisms linking SWM to climate sustainability outcomes, particularly within the resource-constrained institutional context of developing economies like Pakistan. This study steps in precisely at that gap by providing the first empirical test of dual mediation through environmental practices and AI-enabled digitalization in a Pakistani municipal waste management setting, using SEM with bootstrapping to establish both direct and indirect pathways to sustainable development.

The rationale for examining mediation is grounded in established organizational theory. According to the Resource-Based View (RBV), organizations generate superior outcomes by developing valuable, inimitable capabilities (Barney 1991). Environmental practices and AI-enabled digital systems constitute precisely such capabilities, converting raw waste management operations into sustained value. Mediation is theoretically plausible because these capabilities function as intervening mechanisms translating operational inputs (waste collection, segregation, disposal) into sustainability outputs (climate mitigation, resource recovery, social equity) rather than operating as direct antecedents of sustainability. Without institutionalized environmental routines or digital decision-support systems, even well-resourced SWM operations may fail to achieve measurable sustainability results, a process theoretically consistent with mediation rather than moderation (Baron and Kenny 1986; Preacher and Hayes 2008).

This study is anchored in three complementary theoretical perspectives. The Resource-Based View (RBV) proposes that the value of organizational operations can be augmented by strategic capabilities such as environmental management routines and AI-backed digital systems (Barney 1991; Teece 2018). Stakeholder theory emphasizes transparency, accountability, and engagement with diverse actors in managing sustainability challenges, particularly in public service organizations (Freeman et al. 2021). The circular economy framework illustrates the role of waste recovery, reuse, and recycling in transforming waste streams into resource value, contributing to long-term sustainability and climate resilience (Kirchherr et al. 2018). Together, these perspectives constitute a coherent analytical lens through which waste management can be repositioned as a strategic driver of sustainable development.

The novel contribution of this study is threefold. First, it provides the first simultaneous empirical test of environmental practices and AI-enabled digitalization as dual mediating pathways linking SWM to climate sustainability. Second, it integrates the RBV, Stakeholder Theory, and Circular Economy into a single analytical framework applied to operational waste data from a developing economy. Third, it generates contextually relevant, SDG-aligned policy evidence from Pakistan a setting where the intersection of institutional constraint and digital disruption remains underexplored in the sustainability literature.

The research addresses three questions: (1) What is the effect of solid waste management on sustainable development? (2) What is the mediating role of environmental practices and AI-enabled digitalization in this relationship? (3) What theoretical and practical lessons can be derived from integrating waste management, environmental stewardship, and digital innovation for sustainable business models in developing economies?

## Literature Review and Hypotheses Development

### Solid Waste Management and Sustainable Development

Solid waste management (SWM) has been widely recognized as a cornerstone of sustainable development, particularly in urban settings subject to environmental pressure and climate vulnerability (Hoornweg and Bhada-Tata 2012; Kaza et al. 2018). A well-managed waste system contributes to sustainability by reducing pollution, minimizing resource wastage, improving public health, and enhancing economic productivity dimensions consistent with the triple bottom line framework (Elkington 1997; Schroeder et al. 2019). Conversely, inadequately managed waste systems are associated with land and water contamination, greenhouse gas emissions from uncontrolled landfills, health hazards, and the degradation of urban livability (Ferronato and Torretta 2019; Guerrero et al. 2013; Marshall and Farahbakhsh 2013).

In developing economies, the sustainability stakes of SWM are amplified by high rates of urbanization, rapid population growth, and persistent institutional constraints (Zhu et al. 2008; Sujauddin et al. 2008). Pakistan’s major urban centers including Karachi, Lahore, and Peshawar generate millions of tonnes of solid waste annually, yet collection coverage, treatment infrastructure, and governance mechanisms remain critically underdeveloped (Ali et al. 2021; Pervez et al. 2012). Scholars have argued that SWM can no longer be perceived as a routine operational task but must instead be repositioned as a strategic lever aligned with national and international sustainability agendas (Scheinberg et al. 2010; Wilson et al. 2015). Empirical evidence from developing Asian economies supports this repositioning: studies conducted in China (Zhu et al. 2008), Indonesia (Damanhuri and Padmi 2010), and Bangladesh (Islam and Mohamed Romli 2023) demonstrate significant associations between improved SWM systems and measurable sustainability outcomes. However, these studies generally examine direct relationships without investigating the intervening mechanisms specifically environmental governance and digital transformation that condition the strength of this relationship. This gap is precisely what the present study addresses. Based on the above, the following hypothesis is proposed:

**H1:** Solid waste management has a positive and significant impact on sustainable development.

### Environmental Practices as a Mediating Mechanism

Environmental practices encompass the organizational policies, routines, and behaviors that seek to reduce ecological harm and ensure compliance with environmental standards (Molina-Azorín et al. 2009; Sarkis et al. 2010). In the context of municipal services, such practices include pollution prevention programs, waste minimization initiatives, resource use efficiency measures, and formal environmental training for employees (Zhu et al. 2008; Perron et al. 2006). Empirical studies consistently demonstrate that organizations adopting formalized environmental management practices are better positioned to achieve sustainability outcomes (González-Benito and González-Benito 2005; Klassen and Whybark 1999). Environmental management systems (EMS) such as those conforming to ISO 14001 institutionalize ecological responsibility within daily operations, improving regulatory adherence, stakeholder confidence, and service accountability (Morrow and Rondinelli 2002; Nawrocka and Parker 2009).

Critically, several scholars have highlighted a mediating rather than merely direct role for environmental practices. Molina-Azorín et al. (2009) found that environmental management mediates the relationship between organizational strategy and performance across manufacturing sectors. Sarkis et al. (2010) extended this reasoning to supply chain sustainability, showing that environmental routines function as conduits converting operational inputs into measurable ecological outcomes. In the context of public waste services, Guerrero et al. (2013) demonstrated that governance-aligned environmental routines significantly improved household waste diversion rates across 20 cities in lower-middle-income countries. Conversely, Marshall and Farahbakhsh (2013) caution that environmental practices are only effective when institutionalized systematically, as ad hoc compliance efforts fail to produce sustained performance gains. This nuance is relevant to the present study, which examines formalized practices not informal behaviors as potential mediators in Pakistani WSSCs.

**H2:** Environmental practices mediate the relationship between solid waste management and sustainable development.

### AI-Enabled Digitalization as a Mediating Mechanism

Digitalization has emerged as a transformative driver of sustainability by enabling organizations to enhance efficiency, transparency, and data-driven decision-making (Pappas et al. 2018; Vial 2019). Within the context of municipal waste management, AI-enabled digital technologies including intelligent tracking systems, software-based route optimization, electronic service reporting, and performance dashboards support operational planning and systemic control (Abdallah et al. 2020; Al-Thani et al. 2022). These tools reduce inefficiencies, lower operational costs, increase service reliability, and boost stakeholder interaction (Kumar et al. 2023; Lino et al. 2022).

Empirical evidence from smart city implementations confirms the efficiency gains attributable to AI-driven waste management. Al-Thani et al. (2022) reported that GPS-based waste truck tracking in Qatar reduced fuel consumption by 18% and improved collection efficiency. Abdallah et al. (2020) demonstrated that machine learning-based route optimization in Dubai reduced operational costs by up to 14%, while Lino et al. (2022) found that IoT-enabled bin sensors significantly lowered the number of unnecessary collection trips in Portuguese municipalities. However, a crucial distinction exists between digitalization as a direct performance driver and digitalization as a mediating mechanism. Zhang et al. (2020) argue that digital capabilities produce sustainability gains only when they are layered onto sound operational foundations in this case, effective SWM systems. Rosa et al. (2020) similarly found that digital transformation in public service organizations mediated between organizational capacity and sustainability performance, rather than exerting a direct independent effect. This mediation logic justifies treating AI-enabled digitalization as an intervening variable in the present framework, rather than a direct antecedent of sustainability outcomes. Furthermore, in resource-constrained developing economy contexts, digital solutions provide a relatively low-cost pathway to service improvement without requiring large-scale infrastructure investment (Guerrero et al. 2013; Wilson et al. 2015), making them particularly relevant for Pakistani WSSCs.

**H3:** AI-enabled digitalization mediates the relationship between solid waste management and sustainable development.

### Joint Mediation: Environmental Practices and AI-Enabled Digitalization

While the individual mediating effects of environmental practices and AI-enabled digitalization have each received scholarly attention, their combined or synergistic mediating role has not been empirically tested in SWM literature. Theoretical support for joint mediation is strong. From an RBV perspective, the complementarity of organizational capabilities generates greater value than any single capability in isolation (Barney and Clark 2007; Teece 2018). Environmental practices anchor the normative and compliance dimensions of sustainable operations, while digital capabilities provide the informational and technological infrastructure for real-time adaptive management. Together, they create an integrated governance architecture through which the sustainability impacts of SWM are maximized. Stakeholder theory further supports this complementarity: communities and regulators demand both ecological responsibility (addressed by environmental practices) and operational transparency (provided by digital systems) simultaneously (Freeman et al. 2021). Circular economy scholarship adds that material traceability a digital function combined with eco-design and waste minimization environmental functions generates synergistic resource recovery outcomes (Kirchherr et al. 2018; Geissdoerfer et al. 2017). Collectively, these theoretical arguments justify the following hypothesis:

**H4:** The combined mediating effects of environmental practices and AI-enabled digitalization strengthen the positive relationship between solid waste management and sustainable development more than either mediator acting alone.

### Theoretical Framework

Three theoretical perspectives ground this study. First, the Resource-Based View (RBV) postulates that organizations achieve superior performance by building valuable, rare, and inimitable capabilities (Barney 1991). Environmental management routines and AI-facilitated digital systems constitute such capabilities: they are not universally present among municipal operators, they are difficult to imitate in the short term, and they create measurable sustainability value when deployed strategically (Teece 2018; Barney and Clark 2007). Second, Stakeholder Theory emphasizes that organizations must respond to the requirements of governments, communities, employees, and service users (Freeman et al. 2021). Environmental governance and AI-assisted accountability mechanisms make waste management more responsive and legitimate in the eyes of diverse stakeholders, which is critical in settings like Pakistan where public trust in municipal services is chronically low (Ali et al. 2021). Third, the Circular Economy (CE) model frames resource recovery, reuse, and recycling as pathways to long-term sustainability (Kirchherr et al. 2018; Geissdoerfer et al. 2017). Integrating SWM with CE principles enabled by environmental practices that enforce eco-design and by digital systems that enable material traceability can convert waste streams into productive resources, generating circular value while mitigating climate impacts (Ellen MacArthur Foundation 2013).

## Research Methodology

### Research Design and Sampling

**Fig. 1:**
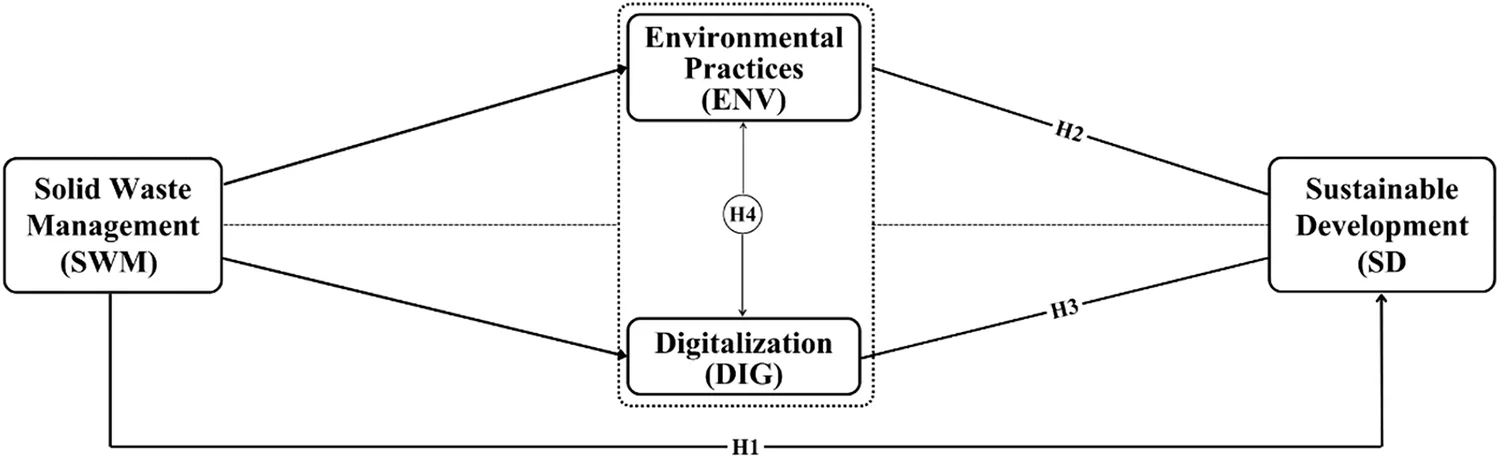
Conceptual framework of the study showing direct and mediating relationships

This study adopts a quantitative, cross-sectional design to empirically investigate the hypothesized relationships among solid waste management, environmental practices, AI-enabled digitalization, and sustainable development (Fig. 1). A quantitative design is appropriate given the need to measure latent constructs systematically and to test theory-driven hypotheses using multivariate statistical methods (Hair et al. 2019). Although cross-sectional designs preclude definitive causal inference, they are widely employed in organizational sustainability research to obtain valid evidence on current practices and outcomes (Podsakoff et al. 2003; Conway and Lance 2010). Structural equation modeling (SEM) with bootstrapped mediation analysis is used to evaluate both the direct path and the indirect pathways through environmental practices and AI-enabled digitalization, following the recommendations of Hair et al. (2022) and Hayes (2018).

The theoretical rationale for each mediated pathway is as follows. The path SWM → Environmental Practices is grounded in the argument that organizations investing in systemic waste management necessarily activate environmental governance routines (Molina-Azorín et al. 2009; Sarkis et al. 2010). The path SWM → AI-Enabled Digitalization reflects the observation that improvements in waste management systems create both the demand and the operational context for deploying smart monitoring and optimization tools (Abdallah et al. 2020; Zhang et al. 2020). Both mediators then independently and jointly transmit their effects to sustainable development outcomes, consistent with the RBV logic of capability complementarity (Barney and Clark 2007).

The target population comprised employees of Water and Sanitation Service Companies (WSSCs) operating in Khyber Pakhtunkhwa, Pakistan. These organizations supply municipal water, sewerage, and solid waste services, constituting a viable institutional context for studying climate-oriented sustainability practices. Seven WSSCs were included to ensure adequate regional coverage.

Sample size was determined following the recommendations of Krejcie and Morgan (1970), which specify a minimum of 384 respondents for large populations. Four hundred and fifty surveys were distributed to account for anticipated non-response. Four hundred and six valid responses were returned and retained for analysis, representing a high response rate attributable to organizational management support and systematic data collection protocols. Stratified random sampling was applied across three organizational strata i.e. managerial, supervisory, and operational staff, to ensure hierarchical representativeness, given that attitudes toward environmental practices and digital systems may vary by role and responsibility level (Sekaran and Bougie 2019).

### Instrumentation and Measurement

Data were collected using a structured questionnaire adapted from validated measurement scales in prior research, ensuring content validity and consistency with existing literature. All items were scored on a five-point Likert scale ranging from 1 (Strongly Disagree) to 5 (Strongly Agree).

Table 1 presents the structured measurement model with construct-level items, scale sources, and reliability statistics. Solid Waste Management (SWM) was measured using eight items adapted from Guerrero et al. (2013) and Ferronato and Torretta (2019), addressing collection efficiency, segregation, recycling, and disposal effectiveness. While Sustainable Development (SD) was assessed via nine items adapted from Schroeder et al. (2019) and Elkington (1997), covering economic efficiency, social responsibility, and environmental protection in accordance with the triple bottom line framework. Environmental Practices (ENV) were measured using six items adapted from Molina-Azorín et al. (2009) and Sarkis et al. (2010), covering regulatory compliance, pollution prevention, employee environmental training, and environmental reporting mechanisms. AI-Enabled Digitalization (DIG) was operationalized using seven items adapted from Abdallah et al. (2020) and Zhang et al. (2020), evaluating the utilization of smart waste tracking, route optimization software, electronic service reporting, and data-driven monitoring systems.

**Table 1:** Structured measurement table constructs, sources, and reliability

Construct	No. of items	Scale source(s)
Solid waste management	8	Guerrero et al. (2013); Ferronato and Torretta (2019)
Sustainable development	9	Schroeder et al. (2019); Elkington (1997)
Environmental practices	6	Molina-Azorín et al. (2009); Sarkis et al. (2010)
AI-enabled digitalization	7	Abdallah et al. (2020); Zhang et al. (2020)

A pilot study involving 30 respondents was conducted prior to full-scale data collection to assess item clarity, relevance, and reliability. Pilot feedback was used to refine item wording and improve overall instrument quality.

### Data Collection and Analysis Procedures

Data collection was conducted between January and April 2025, following formal consent from the management of participating WSSCs. Respondents were informed of the academic purpose of the study and assured of full confidentiality and anonymity to encourage candid responses. Item order was randomized to minimize common method bias (Podsakoff et al. 2003), and respondents were selected across multiple organizational levels to improve representativeness.

Self-administered questionnaires were distributed with trained research assistants available to address clarification requests. Analysis was performed using SPSS and AMOS software and proceeded in three stages. The first stage involved preliminary data screening for missing values, outliers, and normality. Missing data were minimal and addressed via mean imputation. Multivariate outliers were detected and removed using Mahalanobis distance diagnostics. The second stage involved Confirmatory Factor Analysis (CFA) to evaluate the measurement model, assessing construct reliability via Cronbach’s alpha and composite reliability, and convergent and discriminant validity via average variance extracted (AVE) and the Fornell-Larcker criterion (Fornell and Larcker, 1981). The third stage estimated the structural model to test direct and mediated relationships, using a bootstrapping procedure with 5000 resamples and bias-corrected confidence intervals to assess the significance of indirect effects (Hayes 2018; Preacher and Hayes 2008).

## Results and Discussion

### Preliminary Analysis

The dataset was screened prior to hypothesis testing. Of the 450 distributed questionnaires, 406 complete and viable responses were received. Following the removal of three multivariate outliers identified via Mahalanobis distance analysis, the final analytical sample comprised 403 respondents. Missing data were minimal, randomly distributed, and addressed via mean imputation, indicating no systematic bias. Descriptive statistics showed moderate-to-high scores across all constructs, suggesting that municipal employees perceive digital and environmental initiatives as positively influencing operational effectiveness and climate resilience. Normality diagnostics confirmed acceptable skew and kurtosis values, supporting the use of SEM (Hair et al. 2019).

Reliability analysis yielded Cronbach’s alpha and composite reliability values exceeding the recommended threshold of 0.70 for all constructs (Nunnally 1978), and AVE values surpassed the 0.50 minimum criterion for convergent validity (Fornell and Larcker 1981). These statistics are summarized in Table 2.

**Table 2:** Reliability and convergent validity of constructs

Construct	Items	Cronbach’s α	CR	AVE
Solid waste management	8	0.89	0.91	0.62
Sustainable development	9	0.91	0.93	0.65
Environmental practices	6	0.87	0.89	0.59
AI-enabled digitalization	7	0.88	0.90	0.61

All values meet or exceed accepted thresholds (CR > 0.70; AVE > 0.50; Fornell and Larcker 1981).

*CR* Composite Reliability, *AVE* Average Variance Extracted.

### Confirmatory Factor Analysis (CFA)

The measurement model comprising all four constructs was validated via CFA. Table 3 presents model fit was satisfactory: χ²/df = 2.14, CFI = 0.94, TLI = 0.93, RMSEA = 0.051, SRMR = 0.046 all within accepted thresholds (Hu and Bentler 1999). These results confirm that the proposed four-factor structure adequately represents the empirical data.

**Table 3:** Goodness-of-fit indices for the measurement model

Fit index	Recommended threshold	Obtained value
χ²/df	<3.0	2.14
CFI	≥0.90	0.94
TLI	≥0.90	0.93
RMSEA	≤0.08	0.051
SRMR	≤0.08	0.046

All measurement items had factor loadings exceeding 0.60 and were statistically significant (*p* < 0.001), supporting convergent validity. Items related to digital technologies including waste tracking systems, route optimization, and data-driven decision support loaded strongly on the AI-Enabled Digitalization factor, confirming the empirical distinctiveness of this construct from conventional waste management practices. Similarly, environmental practice indicators compliance mechanisms, pollution control, and employee training loaded highly on the Environmental Practices factor.

Discriminant validity was assessed using the Fornell-Larcker criterion (1981), which requires the square root of each construct’s AVE to exceed its correlations with all other constructs. Table 4 reports the inter-construct correlations and AVE square roots on the diagonal, confirming sufficient discriminant validity across all construct pairs.

**Table 4:** Correlations and discriminant validity (diagonal = √AVE)

Construct	SWM	SD	ENV	DIG
Solid waste management	0.79	–	–	–
Sustainable development	0.62	0.81	–	–
Environmental practices	0.55	0.59	0.77	–
AI-enabled digitalization	0.58	0.61	0.54	0.78

### Structural Model and Hypotheses Testing

Structural Equation Modeling (SEM) was used to test the hypothesized direct and mediated relationships. The structural model achieved satisfactory overall fit: χ²/df = 2.25, CFI = 0.93, TLI = 0.92, RMSEA = 0.054. Direct path analysis confirmed that SWM had a positive and statistically significant effect on SD (β = 0.41, *p* < 0.001), supporting H1 as shown in Table 5. This is consistent with findings from Ferronato and Torretta (2019) and Guerrero et al. (2013), who documented significant associations between enhanced SWM system performance and improvements in urban environmental quality and public health. The effect size (β = 0.41) is comparable to that reported by Wilson et al. (2015) in their cross-national study of SWM governance in low- and middle-income cities, reinforcing the external plausibility of the present finding.

**Table 5:** Structural model results direct and mediated paths

Hypothesis	Path	β	*p*-value	Result
H1	SWM → SD	0.41	<0.001	Supported
H2	SWM → ENV → SD	0.13	<0.01	Supported
H3	SWM → DIG → SD	0.12	<0.01	Supported
H4	SWM → ENV + DIG → SD (Joint)	0.21	<0.01	Supported

SWM also exerted a significant effect on Environmental Practices (β = 0.46, *p* < 0.001) and AI-Enabled Digitalization (β = 0.44, *p* < 0.001). Environmental Practices (β = 0.29, *p* < 0.001) and AI-Enabled Digitalization (β = 0.27, *p* < 0.001) each had significant independent effects on SD. Indirect effects analyses confirmed partial mediation through both pathways.

Bootstrapping (5000 resamples, bias-corrected 95% CIs) confirmed the significance of all indirect effects (Hayes 2018; Preacher and Hayes 2008). None of the confidence intervals included zero, confirming partial mediation for both H2 and H3, and joint mediation for H4 (Table 6).

**Table 6:** Bootstrapping mediation results

Mediation path	Indirect effect	95% CI (LL–UL)	Significance
SWM → ENV → SD	0.13	0.07–0.21	Significant
SWM → DIG → SD	0.12	0.06–0.20	Significant
SWM → ENV + DIG → SD	0.21	0.12–0.30	Significant

The partial mediation finding implies that SWM directly contributes to sustainability outcomes, but its impact is substantially augmented when institutionalized environmental governance and smart digital systems are in place. The finding of partial rather than full mediation is theoretically coherent and practically meaningful: waste management systems possess an intrinsic direct sustainability value (e.g., through reduced littering and vector control), independent of governance or digital capabilities, but that value is multiplied when supported by complementary organizational mechanisms consistent with RBV capability complementarity arguments (Barney and Clark 2007).

### Discussion of Findings

This study shows that solid waste management contributes to sustainable development both directly and through environmental practices and AI-enabled digitalization. The findings refine earlier research by demonstrating not only that waste management matters, but also how its sustainability effects are transmitted within municipal organizations.

The significant direct effect of solid waste management on sustainable development confirms that core operational improvements retain intrinsic sustainability value. Better collection, segregation, and disposal can reduce pollution and strengthen public health outcomes even before environmental governance and digital systems are added. At the same time, the persistence of this direct path after introducing the mediators indicates that digital tools should not be treated as substitutes for sound waste operations.

The mediation findings show that environmental practices and AI-enabled digitalization function as two complementary channels. In the present sample, the indirect effects are similar in magnitude, which suggests that formal governance routines remain as important as digital adoption in Pakistan’s WSSCs. This is a meaningful contextual result. In resource-constrained municipal settings, regulatorily embedded environmental practices may offer a more immediate and dependable route to sustainability gains, while digital systems can deepen and scale those gains when supporting infrastructure and staff capacity are available.

The joint mediation result is the strongest effect in the model. This indicates that municipalities gain the greatest benefit when they combine environmental governance with digital intelligence rather than investing in one domain alone. The finding aligns with the Resource-Based View because complementary capabilities create more value together. It also supports stakeholder and circular economy perspectives by linking transparency, compliance, resource efficiency, and traceability within one governance system.

The findings also have clear SDG relevance. Improved waste systems and digital monitoring support SDG 11 by strengthening urban service quality. Circular economy practices enabled by environmental and digital capabilities support SDG 12. Better waste diversion and more organized collection practices can contribute to SDG 13 by reducing climate-related impacts associated with unmanaged waste systems.

These results lead directly to the practical measures outlined in the next section. Because the strongest sustainability gains arise when operational waste management is combined with formal environmental routines and targeted digital systems, the policy measures below translate the empirical findings into implementable actions for governments, municipal operators, and technology providers.

## Policy Implications and Recommendations

### Policy Action for Municipal and National Policymakers

Technology providers should adapt digital solutions and actions to the institutional and infrastructural realities of municipal Pakistan (Table 7). The goal is not technological sophistication alone. The goal is practical usability within low-resource public service environments.

**Table 7:** Policy action for municipal and national policy makers

S.No	Action	Brief rationale
1	Align solid waste management with SDG reporting frameworks	Links waste diversion, recycling, and emission reduction indicators to SDGs 11, 12, and 13.
2	Create performance-linked incentives for dual investment	Rewards municipalities that combine environmental governance measures with digital monitoring systems.
3	Set national minimum standards for AI-based waste monitoring	Improves procurement consistency and baseline technical compatibility across WSSCs.

### Action for Institutional and Organizational Administrators

Organizational administrators should focus on strengthening formal routines and actions that connect daily waste operations to sustainability outcomes (Table 8). Environmental practices are not peripheral to performance. They are one of the core channels through which waste management produces measurable climate and sustainability benefits.

**Table 8:** Action for institutional and organizational administrators

S.No	Action	Brief rationale
1	Institutionalize quarterly environmental training	Builds role-specific capacity in pollution prevention, waste segregation, and climate-related performance standards.
2	Run semi-annual compliance audits	Improves accountability, supports continuous improvement, and strengthens public trust.
3	Adopt digital tools in phased modules	Reduces implementation risk by starting with GPS tracking and basic reporting before advanced AI systems.

### Action for Technology Developers and AI Solution Providers

Technology providers should adapt digital solutions and actions to the institutional and infrastructural realities of municipal Pakistan (Table 9). The goal is not technological sophistication alone. The goal is practical usability within low-resource public service environments.

**Table 9:** Action for technology developers and AI solution providers

S.No	Action	Brief rationale
1	Design low-bandwidth, offline-capable tools	Improves usability in areas with uneven connectivity and limited ICT infrastructure.
2	Build interoperability with compliance systems	Reduces reporting burden and links digital systems to environmental governance routines.
3	Include citizen-facing transparency dashboards	Strengthens accountability, complaint tracking, and public confidence in municipal services.

## Conclusion, Limitations, and Future Research

This study finds that solid waste management improves sustainable development in Pakistani municipal services and that this effect becomes stronger when environmental practices and AI-enabled digitalization are integrated into routine operations. The direct effect of solid waste management remains important, but the combined mediation pathway is strongest. The central implication is clear: municipalities achieve better climate and sustainability outcomes when they pair operational waste improvements with formal environmental governance and practical digital systems.

Study contributes to literature in three ways. It provides a dual mediation test in a developing-economy municipal context, integrates three complementary theoretical perspectives within one explanatory framework, and offers policy-relevant evidence from an underexplored South Asian setting.

Several limitations should be acknowledged. The cross-sectional design does not permit causal inference. The sample is limited to WSSCs in Khyber Pakhtunkhwa, which constrains generalizability. The use of self-reported perceptual data may introduce common method bias despite procedural safeguards. The study also captures only employee perspectives and therefore does not include the views of citizens, regulators, or civil society actors.

Future research should address these limitations through longitudinal designs, comparative studies across provinces or countries, and expanded models that test moderating conditions such as leadership style, organizational culture, financial capacity, and donor support. Studies should also examine emerging technologies such as IoT-connected bins, blockchain-based traceability, and satellite-assisted waste monitoring. Research that incorporates citizen participation and community co-production would offer a fuller account of how sustainability transitions unfold in municipal waste systems.

## Data Availability

No datasets were generated or analyzed during the current study.
